# Supplementary material for: Lignans Extract from Knotwood of Norway Spruce—A Possible New Weapon against GTDs
Source: J Fungi (Basel). 2022 Mar 30;8(4):357. doi: 10.3390/jof8040357 (PMC9025846; doi:10.3390/jof8040357)
Supplement: Supplementary file 1 [file jof-08-00357-s001.zip › Figure S1.pdf]

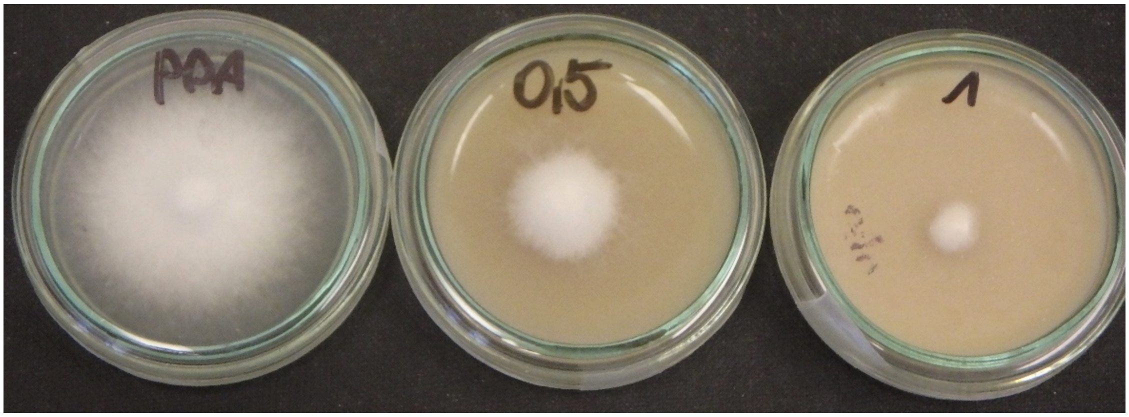


**Figure S1**. *Eutypa lata (*strain MEND-F-0015) on PDA plates supplemented with GE five days after incubation. Numbers on plates represent different concentrations of HMR in medium, from the left: control, 0.5 and 1 mg. $ml^{-1}$.
