# Supplementary material for: Lignans Extract from Knotwood of Norway Spruce—A Possible New Weapon against GTDs
Source: J Fungi (Basel). 2022 Mar 30;8(4):357. doi: 10.3390/jof8040357 (PMC9025846; doi:10.3390/jof8040357)
Supplement: Supplementary file 1 [file jof-08-00357-s001.zip › Figure S4.pdf]

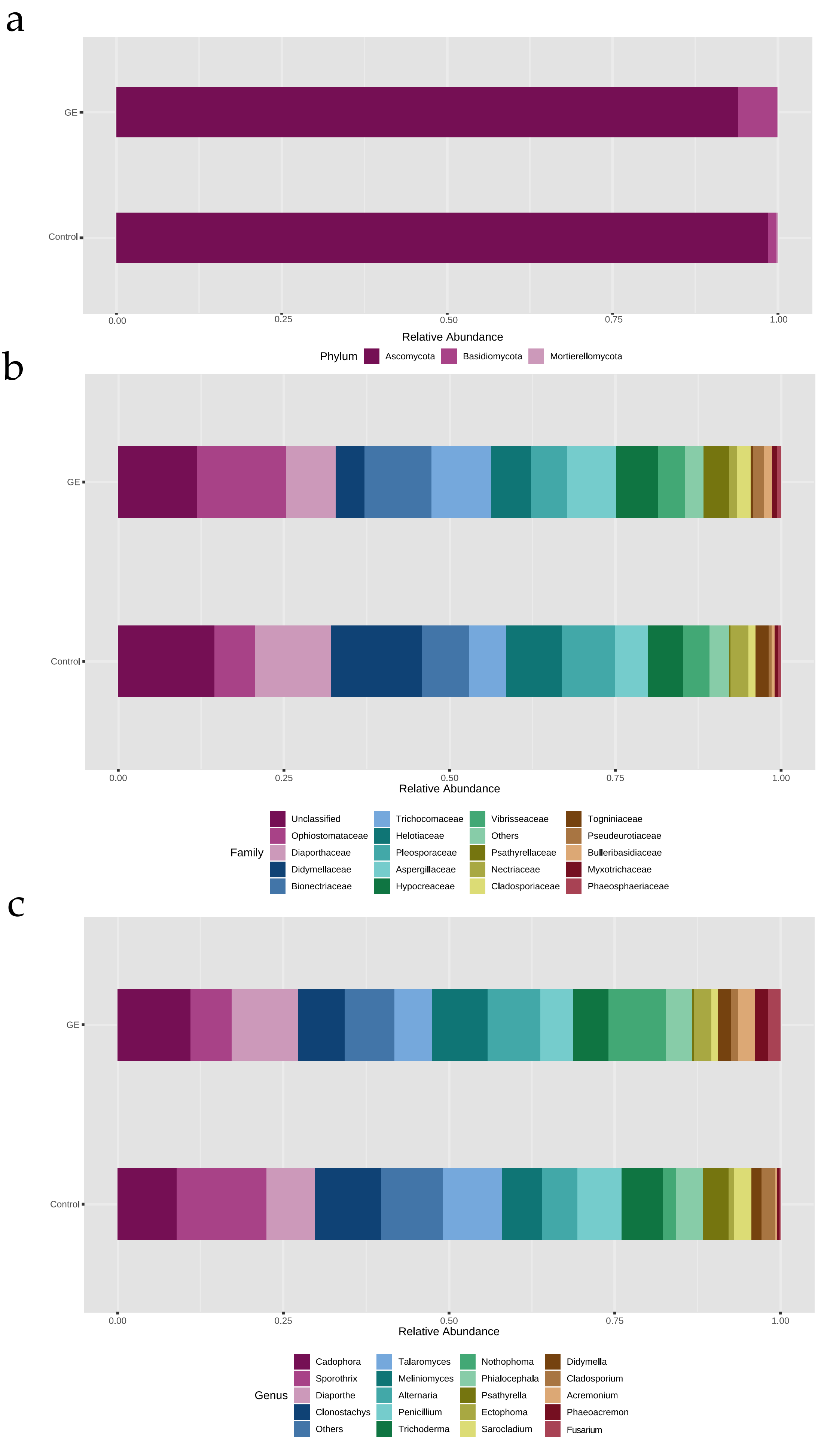
a
GE
Control
0.00
0.25
0.50
0.75
1.00
Relative Abundance
Phylum
Ascomycota
Basidiomycota
Mortierellomycota
b
GE
Control
0.00
0.25
0.50
0.75
1.00
Relative Abundance
Family
Unclassified
Ophiostomataceae
Diaporthaceae
Didymellaceae
Bionectriaceae
Trichocomaceae
Helotiaceae
Pleosporaceae
Aspergillaceae
Hypocreaceae
Vibrisseaceae
Others
Psathyrellaceae
Nectriaceae
Cladosporiaceae
Togniniaceae
Pseudeurotiaceae
Bulleribasidiaceae
Myxotrichaceae
Phaeosphaeriaceae
c
GE
Control
0.00
0.25
0.50
0.75
1.00
Relative Abundance
Genus
Cadophora
Sporothrix
Diaporthe
Clonostachys
Others
Talaromyces
Meliniomyces
Alternaria
Penicillium
Trichoderma
Nothophoma
Phialocephala
Psathyrella
Ectophoma
Sarocladium
Didymella
Cladosporium
Acremonium
Phaeoacremon
Fusarium
