# Supplementary material for: Lignans Extract from Knotwood of Norway Spruce—A Possible New Weapon against GTDs
Source: J Fungi (Basel). 2022 Mar 30;8(4):357. doi: 10.3390/jof8040357 (PMC9025846; doi:10.3390/jof8040357)
Supplement: Supplementary file 1 [file jof-08-00357-s001.zip › List of supplementary files.pdf]

Figure S1. *Eutypa lata (*strain MEND-F-0015) on PDA plates supplemented with GE five days after incubation. Numbers on plates represent different concentrations of HMR in medium, from the left: control, 0.5 and 1 mg. ml^{-1}.

Figure S2. List of sequenced samples with the number of reads per sample.

Figure S3. Rarefaction curves for each sample.

Figure S4. Relative abundances of different fungal **(a.)** phyla **(b.)** families **(c.)** genera; dataset combining both sampling periods, treated plants (GE) vs. untreated plants.

Figure S5. Alpha diversity metrics combining both sampling periods, treated plants (GE) vs. untreated plants.

Figure S6. Prevalence of different fungal OTUs within samples **(a.)** untreated plants, 10 days after treatment **(b.)** plants treated with GE 10 days after treatment **(c.)** untreated plants, 180 days after treatment **(d.)** plants treated with GE 180 days after treatment.

Figure S7. Detailed LEfSe analyses discriminating significantly different OTUs between treated plants (GE) × untreated plants **(a)**. *Cadophora* 180 days after treatment; **(b)**. *Diaporthe* 10 days after treatment; **(c)**. *Phaeoacremonium* 10 days after treatment.

Table S1. Inhibition effects of GE on the growth of GTD fungi *in vitro* [%] after five days of incubation for all fungi except *C. luteo-olivacea* and *P. minimum,* which were measured after 10 days.

Table S2. Comparison of inhibition effects of GE vs. HMR™ on the growth of GTD fungi *in vitro* [%] after five days of incubation for all fungi except *C. luteo-olivacea* and *P. minimum,* which were measured after 10 days.

Table S3. Good's coverage, Chao1 and Shannon alpha diversity metrics for all samples.

Table S4. OTU sharing between both treatments and periods.

Table S5. OTUs that were unique for each treatment and sampling term.

Table S6. OTU Table.
