# Supplementary material for: Lignans Extract from Knotwood of Norway Spruce—A Possible New Weapon against GTDs
Source: J Fungi (Basel). 2022 Mar 30;8(4):357. doi: 10.3390/jof8040357 (PMC9025846; doi:10.3390/jof8040357)
Supplement: Supplementary file 1 [file jof-08-00357-s001.zip › Table S1.pdf]

	concentration of HMR in GE plates [mg.ml^{-1}]		
**Fungus**	**0.1**	**0.5**	**1**
C. luteo-olivacea	25.8	41.7	100
Da. torresensis	30.7	65.3	100
D. ampelina	47.5	60	62.5
D. bohemiae	32.4	55.8	58.4
Dip. seriata	0	27.5	30
E. lata	32.8	60.8	79
P. minimum	0	26.6	100

Table S1. Inhibition effects of GE on the growth of GTD fungi *in vitro* [%] after five days of incubation for all fungi except *C. luteo-olivacea* and *P. minimum,* which were measured after 10 days.
