# Supplementary material for: Lignans Extract from Knotwood of Norway Spruce—A Possible New Weapon against GTDs
Source: J Fungi (Basel). 2022 Mar 30;8(4):357. doi: 10.3390/jof8040357 (PMC9025846; doi:10.3390/jof8040357)
Supplement: Supplementary file 1 [file jof-08-00357-s001.zip › Table S2.pdf]

**Fungus**	**GE** [1 mg.ml HMR]	**HMR™** [1 mg.ml HMR]
C. luteo-olivacea	100	47
Da. torresensis	100	71.1
D. ampelina	62.5	67.5
D. bohemiae	58.4	58.1
Dip. seriata	30	5
E. lata	79	72
P. minimum	100	26.6

Table S1. Comparison of inhibition effects of GE vs. HMR™ on the growth of GTD fungi *in vitro* [%] after five days of incubation for all fungi except *C. luteo-olivacea* and *P. minimum,* which were measured after 10 days.
