# Supplementary material for: Lignans Extract from Knotwood of Norway Spruce—A Possible New Weapon against GTDs
Source: J Fungi (Basel). 2022 Mar 30;8(4):357. doi: 10.3390/jof8040357 (PMC9025846; doi:10.3390/jof8040357)
Supplement: Supplementary file 1 [file jof-08-00357-s001.zip › Table S5.pdf]

**Control 10-days**	**GE 10-days**	**Control 180-days**	**GE 180-days**
Sigarispora	*Typhula*	*Cephalotrichum*	*Cuniculitrema*
Epicoccum	*Lopadostoma*	*Umbelopsis*	*Cystobasidium*
Wojnowiciella	*Diatrypella*	*Diplodia*	*Camarosporioides*
Spumatoria	*Rachicladosporium*	*Veronaea*	*Pichia*
Belonopsis	*Pleiochaeta*	*Coprinellus*	*Monilia*
Myriangium	*Curvularia*	*Pseudeurotium*	*Periconia*
Plenodomus	*Dictyosporium*	*Sistotrema*	*Aquilomyces*
Cercophora	*Trematosphaeria*	*Bipolaris*	*Phaeomollisia*
Lyomyces	*Rhynchosporium*	*Cosmospora*	*Lasiodiplodia*
Sphaerulina	*Minimelanolocus*	*Sporisorium*	*Dimorphosporicola*
Ochrocladosporium	*Vermiconia*	*Microascus*	*Serendipita*
Coniosporium	*Bionectria*	*Hirsutella*	*Parapenidiella*
Macrophoma	*Mycena*	*Leptodontidium*	*Septoria*
Helicodendron	*Katumotoa*	*Buckleyzyma*	*Acrostalagmus*
Naganishia	*Collariella*	*Teichospora*	*Leucaenicola*
Cylindrocladiella	*Melanocarpus*	*Taphrina*	*Thelonectria*
Allophoma	*Preussia*	*Harposporium*	*Quambalaria*
Tubeufia	*Stagonospora*	*Dothiora*	*Coprinopsis*
Stachybotrys	*Robillarda*	*Radulidium*	*Amorphotheca*
Cystofilobasidium	*Fuscostagonospora*	*Neostagonospora*	*Dialonectria*
Populocrescentia	*Pleurostoma*	*Umbilicaria*	*Phialemonium*
	Humicola	*Papiliotrema*	*Peniophora*
	Allantophomopsiella	*Moesziomyces*	*Neoascochyta*
	Chromelosporium	*Skyttea*	*Holtermanniella*
	Hermatomyces	*Xenasmatella*	*Ophiostoma*
		Fusicolla	*Xenoramularia*
		Clitopilus	*Pyrenochaetopsis*
		Devriesia	*Libertasomyces*
		Monocillium	*Phaeosphaeria*
			Pleopsidium
			Pseudocosmospora
			Nemania
			Cylindrocarpon
			Melanocucurbitaria
			Didymocrea
			Microsporum
			Extremus
			Quadricrura
			Nigrograna
			Volutella
