# Supplementary figures and images for: Lignans Extract from Knotwood of Norway Spruce—A Possible New Weapon against GTDs
Source: J Fungi (Basel). 2022 Mar 30;8(4):357. doi: 10.3390/jof8040357 (PMC9025846; doi:10.3390/jof8040357)

# Library Size Overview

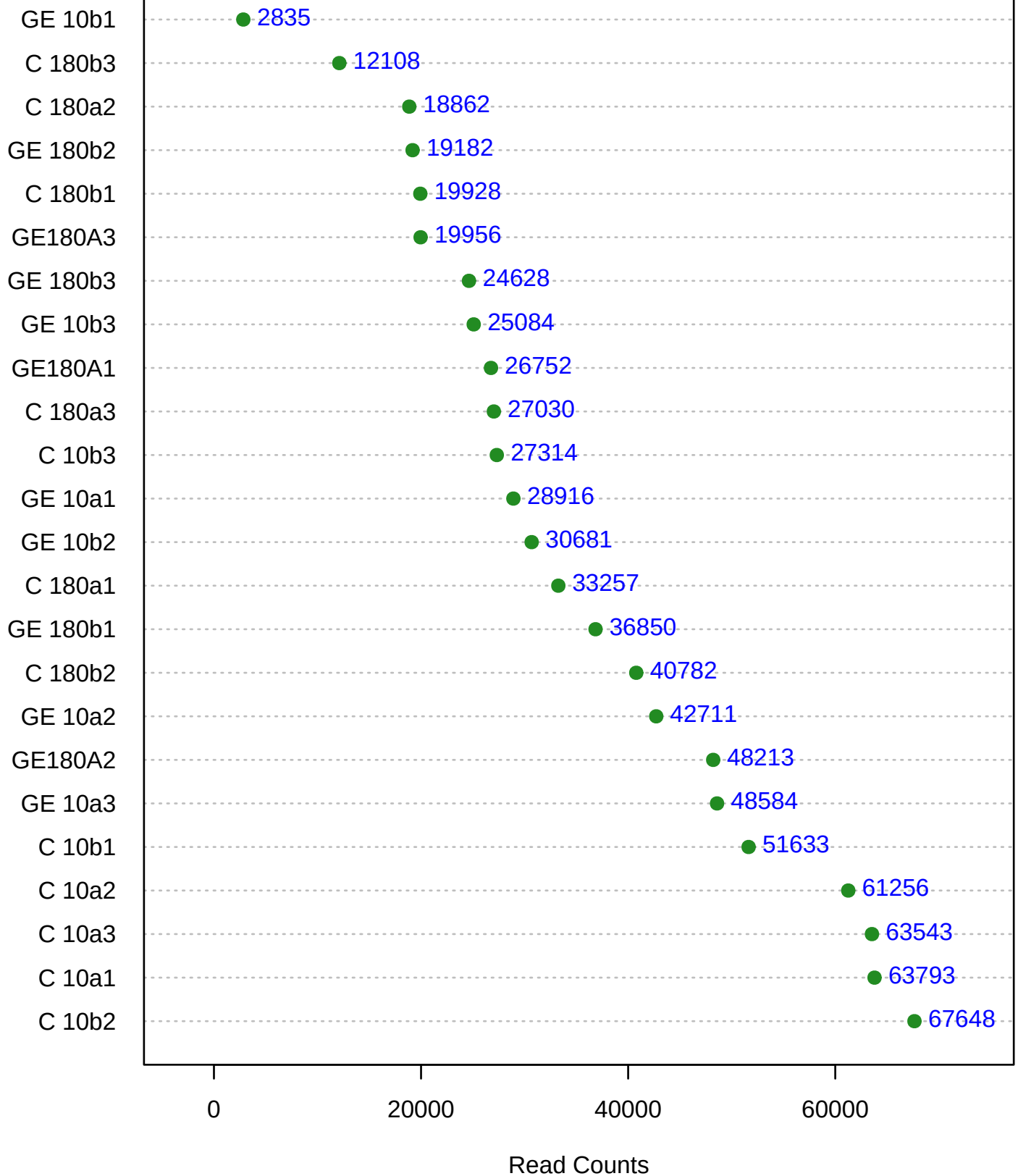

Supplement: Supplementary file 1 [file jof-08-00357-s001.zip › Figure S2.pdf]

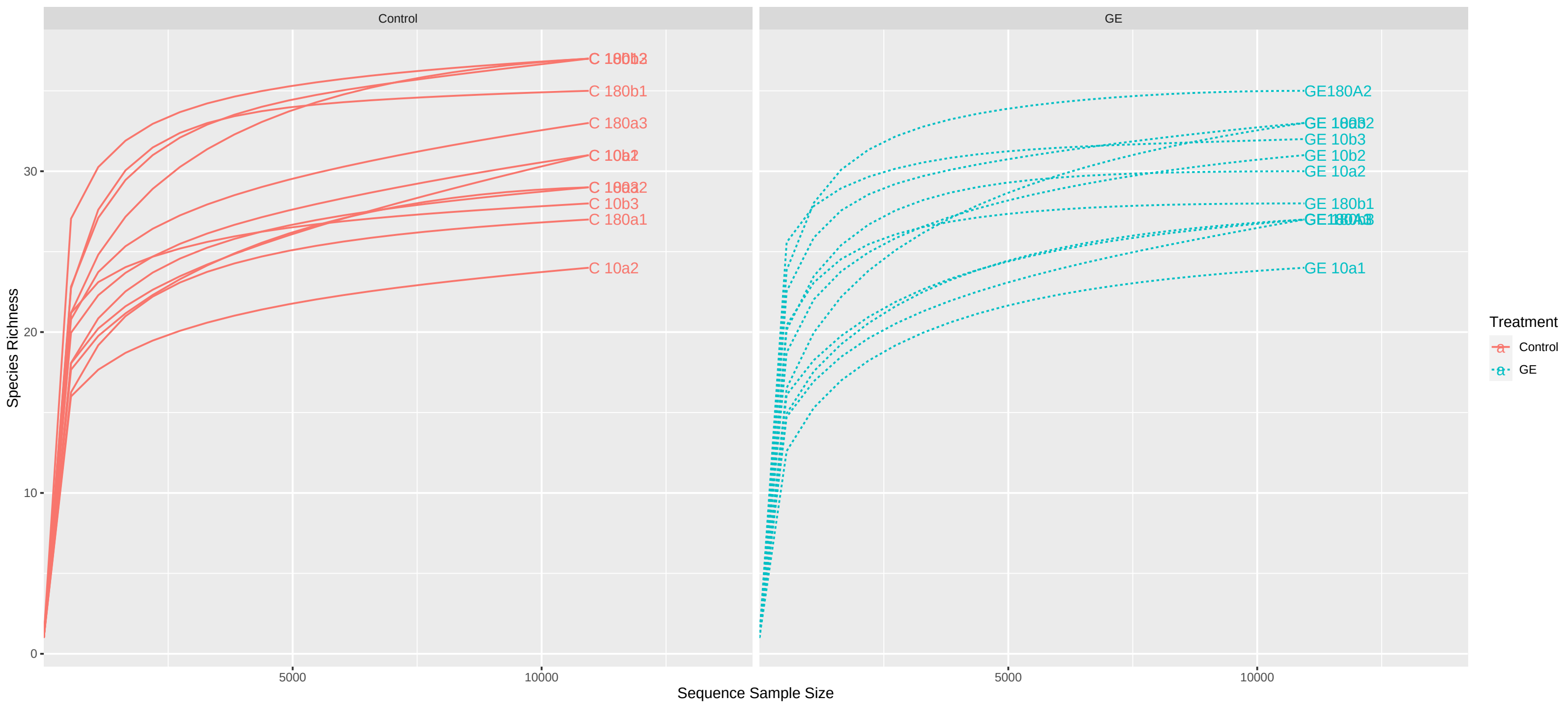

Supplement: Supplementary file 1 [file jof-08-00357-s001.zip › Figure S3.pdf]

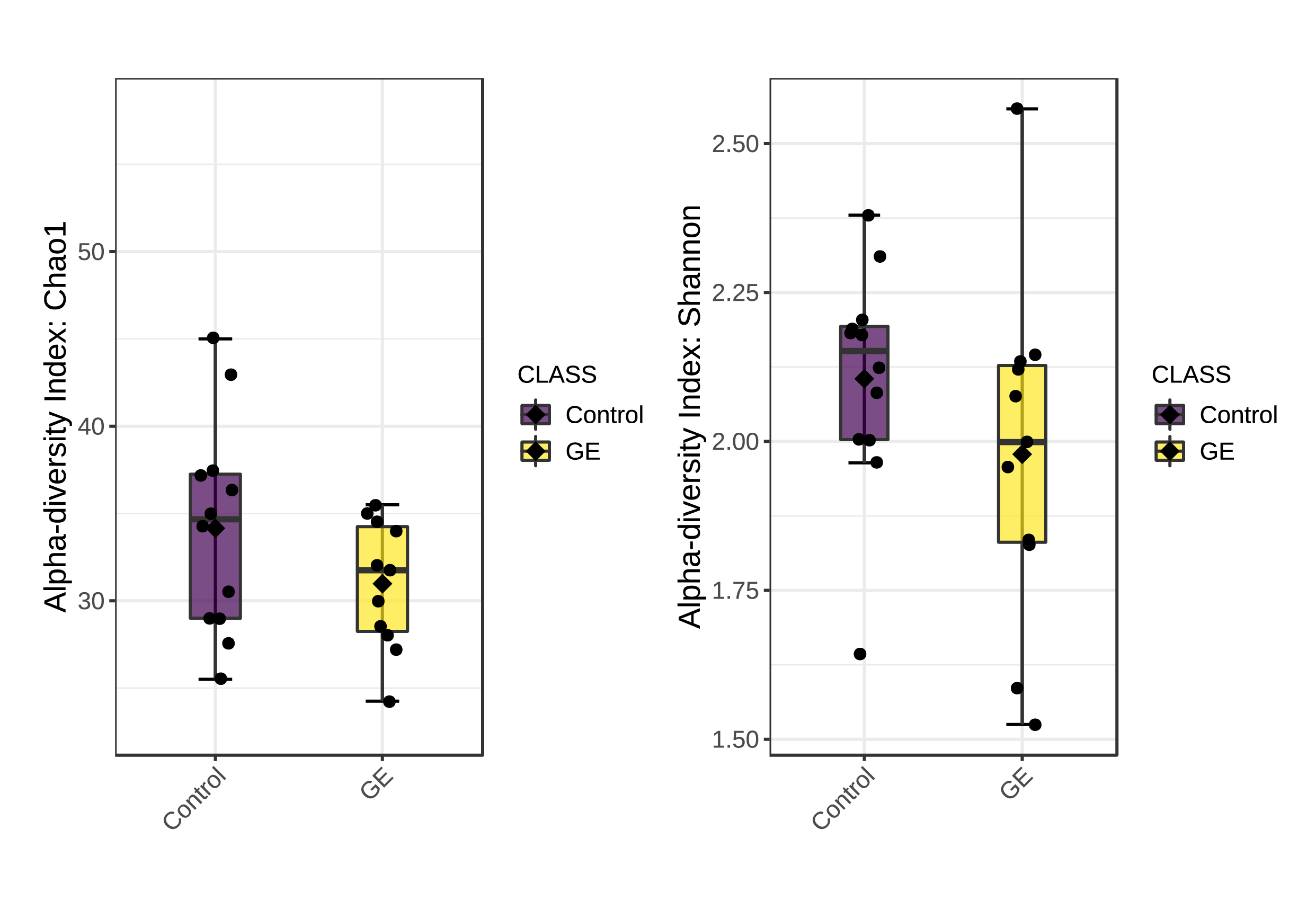

Supplement: Supplementary file 1 [file jof-08-00357-s001.zip › Figure S5.jpg]

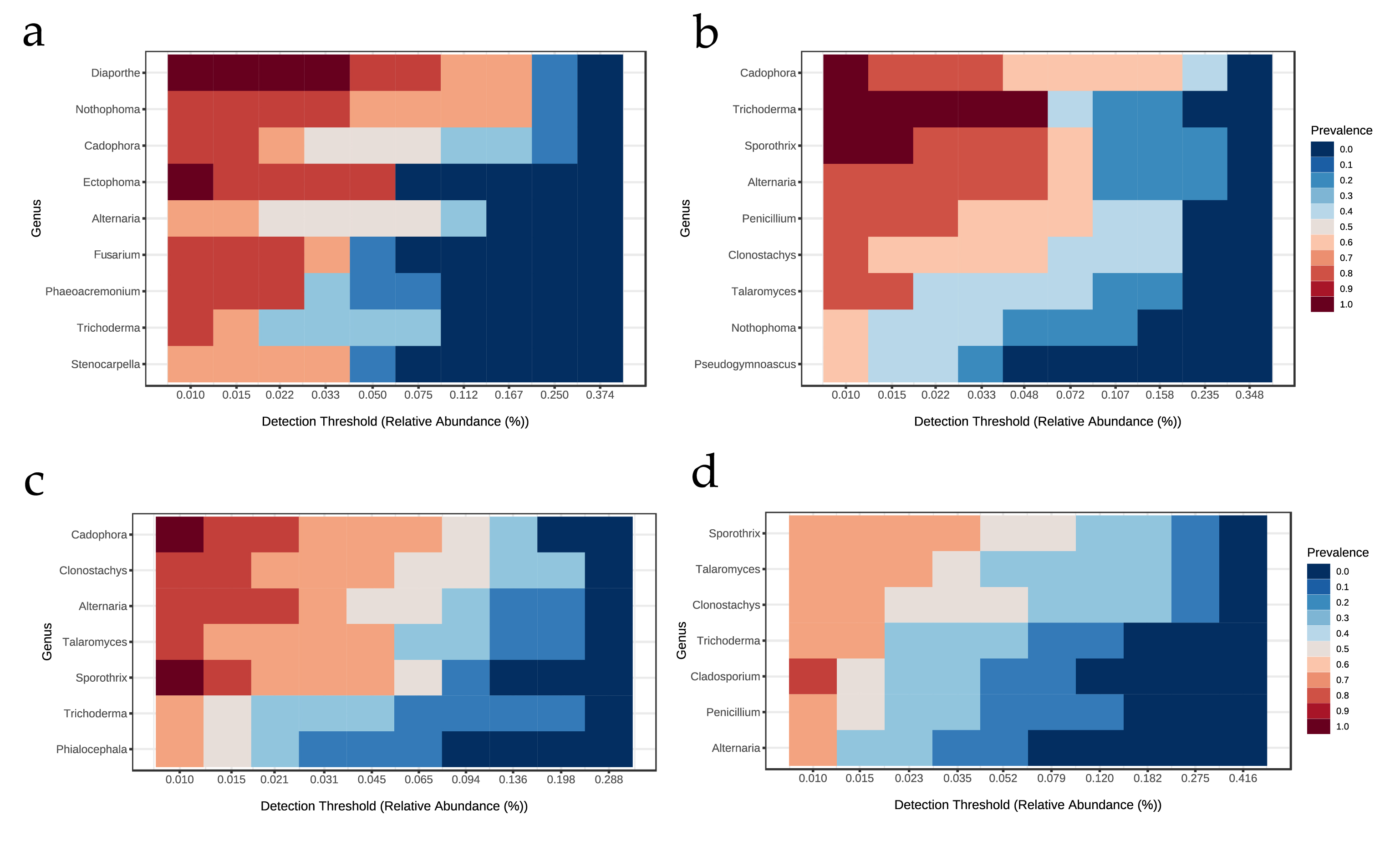

Supplement: Supplementary file 1 [file jof-08-00357-s001.zip › Figure S6.jpg]

a

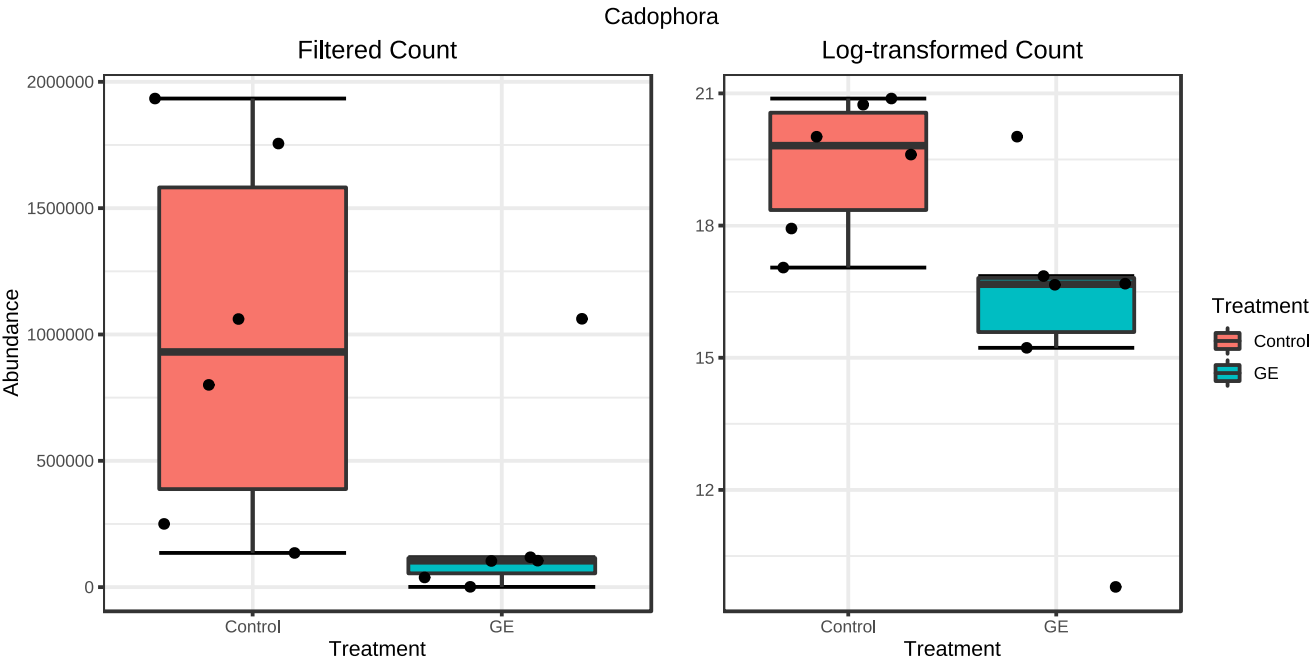

b

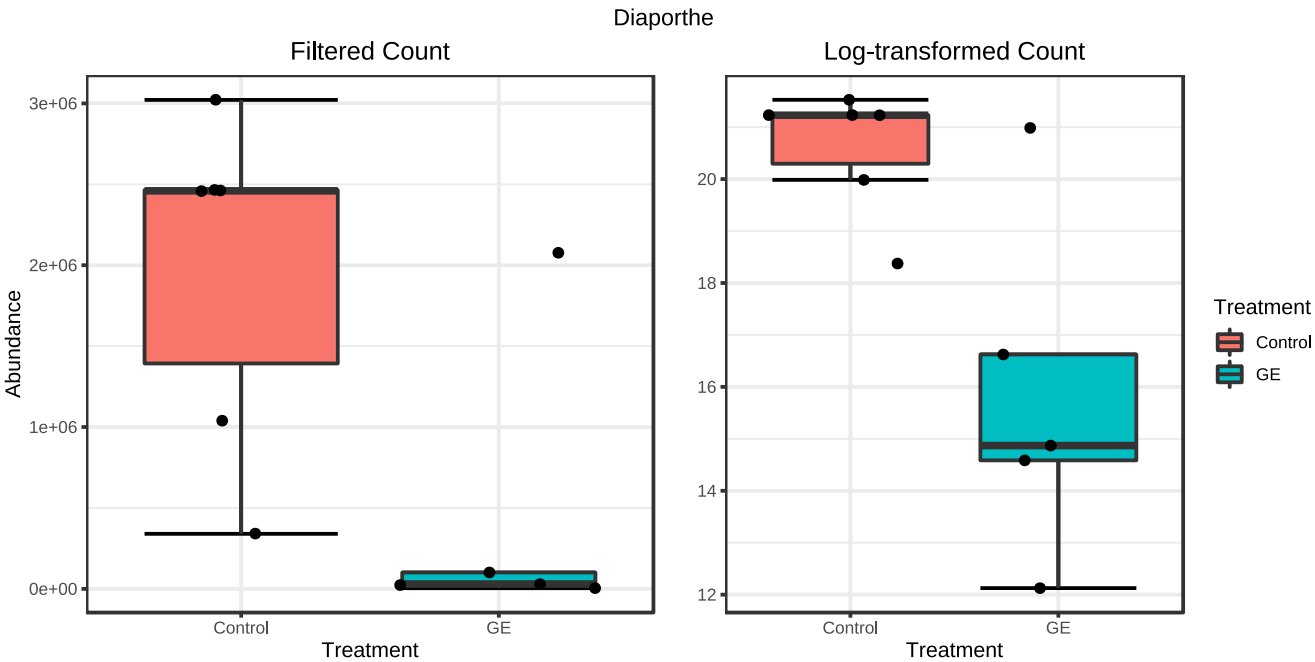

c

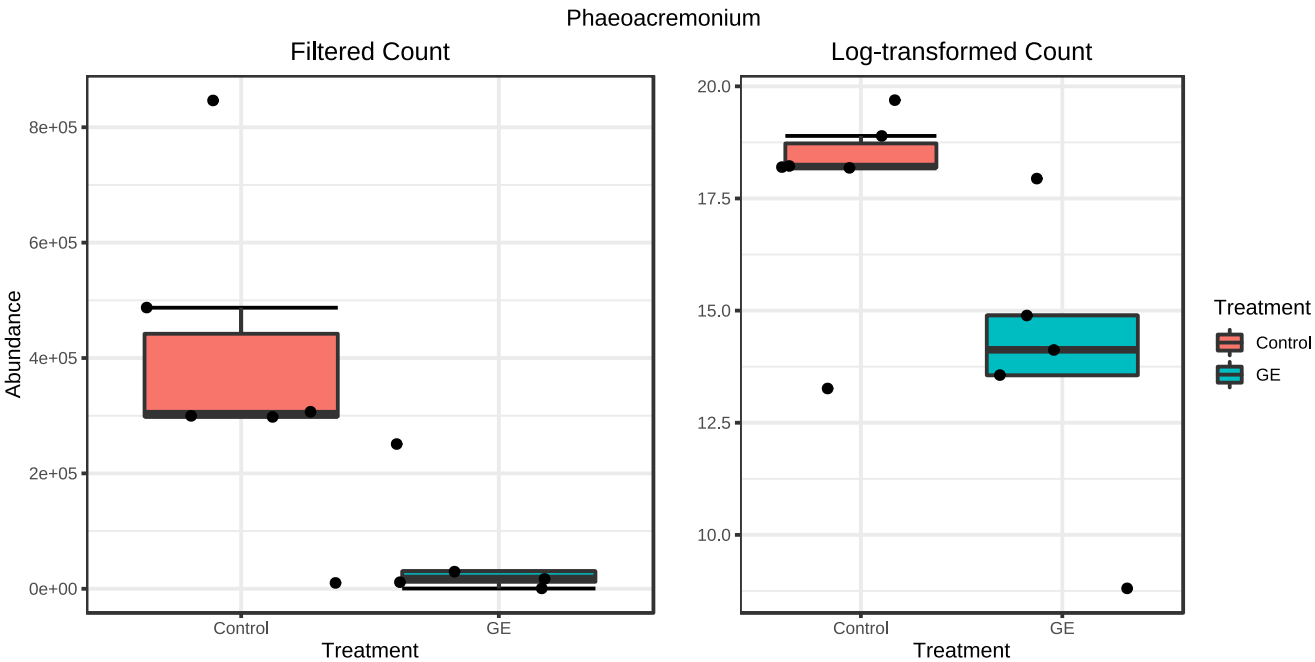

Supplement: Supplementary file 1 [file jof-08-00357-s001.zip › Figure S7.pdf]
